# Coordination of voice, hands and feet in rhythm and beat performance

**DOI:** 10.1038/s41598-022-11783-8

**Published:** 2022-05-16

**Authors:** Signe Hagner Mårup, Cecilie Møller, Peter Vuust

**Affiliations:** 1 Center for Music in the Brain, Department of Clinical Medicine, Aarhus University & The Royal Academy of Music Aarhus/Aalborg, Aarhus, Denmark; 2 Department of Dramaturgy and Musicology, Institute of Communication and Culture, Aarhus University, Aarhus, Denmark

**Keywords:** Human behaviour, Motor control

## Abstract

Interlimb coordination is critical to the successful performance of simple activities in everyday life and it depends on precisely timed perception–action coupling. This is particularly true in music-making, where performers often use body-movements to keep the beat while playing more complex rhythmic patterns. In the current study, we used a musical rhythmic paradigm of simultaneous rhythm/beat performance to examine how interlimb coordination between voice, hands and feet is influenced by the inherent figure-ground relationship between rhythm and beat. Sixty right-handed participants—professional musicians, amateur musicians and non-musicians—performed three short rhythmic patterns while keeping the underlying beat, using 12 different combinations of voice, hands and feet. Results revealed a bodily hierarchy with five levels (1) left foot, (2) right foot, (3) left hand, (4) right hand, (5) voice, i.e., more precise task execution was observed when the rhythm was performed with an effector occupying a higher level in the hierarchy than the effector keeping the beat. The notion of a bodily hierarchy implies that the role assigned to the different effectors is key to successful interlimb coordination: the performance level of a specific effector combination differs considerably, depending on which effector holds the supporting role of the beat and which effector holds the conducting role of the rhythm. Although performance generally increased with expertise, the evidence of the hierarchy was consistent in all three expertise groups. The effects of expertise further highlight how perception influences action. We discuss the possibility that musicians’ more robust metrical prediction models make it easier for musicians to attenuate prediction errors than non-musicians. Overall, the study suggests a comprehensive bodily hierarchy, showing how interlimb coordination is influenced by hierarchical principles in both perception and action.

## Introduction

Interlimb coordination is critical to the successful performance of simple activities in everyday life, such as walking or performing bimanual tasks. Such activities depend on precisely timed perception–action coupling^1^. In many cases, interlimb coordination is also guided by a conduct-support relationship between the effectors, mirroring the inherent conduct-support relationship of an integrated task. For instance, the primary goal of peeling an apple is realized by the dominant hand while the non-dominant hand holds the supporting role of holding the apple^2^. This horizontal dimension characteristic of bimanual interlimb coordination has been extensively studied^2–4^. In the present study, we examined how the conduct-support relationship of an integrated musical task influences human interlimb coordination, also in the vertical dimension. Through the use of a musical rhythm paradigm, we utilized the inherent figure-ground relationship between rhythm and beat^5^ to assign conductive and supportive roles to separate effectors, i.e., feet, hands and voice (in congruent and incongruent combinations). This enabled us to provide a comprehensive account of how interlimb coordination is governed by hierarchical principles present in both action and perception. Please note that although the voice is not considered a 'limb' in a strict sense, we use the term 'interlimb coordination' to also encompass voice in the current paper.

Music is particularly well-suited for studying timing-related aspects of perception/action coupling^6^. When listening to music, we automatically extract rhythmic regularities, most saliently the underlying beat, and we group and nest these regularities to form a hierarchical metrical structure that is used as a framework for interpreting the perceived sound events^5,7–11^. Body movements like foot-tapping or nodding play a facilitatory role in extracting regular beats from complex musical rhythms^12^. Expressed in the figure-ground terminology of the Gestalt tradition^13^, body movements reinforce the perceptual metric ground upon which the rhythmic figure is interpreted. Rhythm perception has recently been linked to theories posing that music perception and action rest on the human brain’s fundamental capacity for prediction^14^. In this view, the metrical structure forms a predictive model that is constantly shaped and challenged by produced or auditorily perceived rhythms. As such, when playing or listening to music, synchronizing body movements to the beat underlines and reinforces prediction, and thereby facilitates reduction of prediction errors^15^.

Synchronizing body movement to musical beats depends on the unique human ability to extract regular beats from complex rhythms. It is a trait that appears to be innate in humans^16^—or in any case develops spontaneously—and tapping along with a beat is relatively easy for most people, musically trained or not^17–20^. A considerable amount of the research on sensorimotor synchronization is represented by tapping studies in which participants were asked to tap with either a rhythm or its underlying beat^12,21–24^. In comparison, little is known about simultaneous performance of beat and rhythm. One study^25^ showed that simultaneous performance of rhythm and beat with combinations of hands and feet is governed by a so-called “order of rhythm dominance”. This corresponds to a bodily hierarchy consisting of four levels: (1) left foot, (2) right foot, (3) left hand, (4) right hand, where right-handed participants prefer to perform the rhythm with an effector occupying a higher level in the hierarchy than the effector keeping the beat. The hierarchy implies different levels of difficulty in the coordination of two effectors, depending on whether their respective roles of ‘rhythm performer’ and ‘beat keeper’ are in accordance with or go against the bodily hierarchy. The study also found effects of musical expertise, where coordinating two effectors in simultaneous rhythm and beat production was more difficult for non-musicians than for musicians.

Importantly, the hierarchical organization of effectors does not imply that each effector has an inherent preference for performing either the rhythm or the beat; this preference changes depending on which effector performs the opposite role. In his work on the development of motor dexterity, Bernstein^26^ distinguishes between four levels of movements (A, B, C and D), which developed through evolution, with each new level represented in a new brain structure and representing the previously inaccessible solution to a new class of motor problems. He makes a distinction between *body dexterity*, which is controlled at Level B (the level of synergy) and Level C (the level of space), and *hand or object dexterity*, which is primarily controlled at Level D (the level of actions), and supported by subordinate levels (A, B and C). Bernstein's hand or object dexterity may be comparable to what we will refer to as “single-effector dexterity”, while body dexterity highlights the bodily coordination which is essentially the kind of dexterity that this study seeks to quantify.

**Figure 1:**
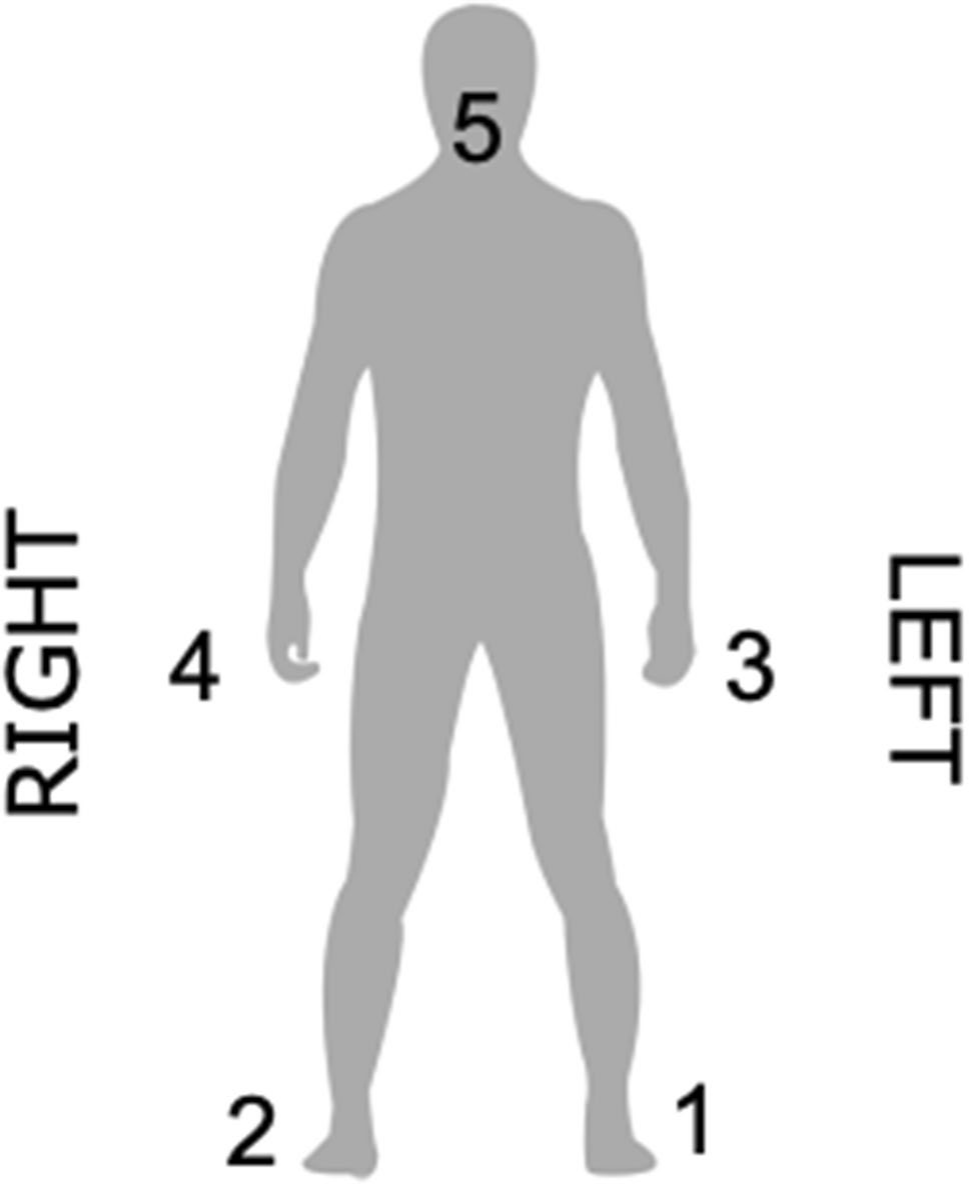
Elements of the proposed bodily hierarchy for right-handed people. (5) voice, (4) right hand, (3) left hand, (2) right foot, (1) left foot. The figure was created using Adobe Illustrator 2018 22.0.0 (https://www.adobe.com/products/illustrator.html) and Microsoft Word for Mac, version 16.49 (21050901) (https://www.microsoft.com/da-dk/microsoft-365/word).

In music, the voice is perhaps the most versatile means to produce sound and rhythm. As a case in point, vocalizing for instance the rhythm of the famous musical phrase “We’re Sergeant Pepper’s Lonely Hearts Club Band” (The Beatles, 1969) while clapping in time with the underlying beat is markedly easier than reversing the roles of the hands and the voice, i.e., vocalizing a regular beat (da-da-da-da) while clapping the more complex rhythm. Therefore, we propose a comprehensive bodily hierarchy consisting of both voice, hands and feet (Fig. 1). Several studies on verbal and manual coordination exist, but they primarily investigated production of sentences or very simple, repetitive rhythmic patterns, often with the hands tapping as fast as possible^27–31^. In our study, the inherent hierarchical properties of rhythm and beat creates a coordination task where the two components of an integrated task—performing a rhythm and keeping a beat, respectively—can be performed on equal terms by both voice, hands and feet. This enables us to study the differences between moving with or against the proposed hierarchy.

To assess whether this bodily hierarchy reflects the hierarchical structure of rhythms in music, i.e., with lower levels in the bodily hierarchy representing lower levels in the metrical structure and vice versa, we asked participants to use their bodies as a musical instrument. We adapted and refined the basic paradigm used by Ibbotson and Morton^25^. Professional musicians, amateur musicians and non-musicians were included in order to assess putative effects of expertise, as well as a second measure of musical competence (The Musical Ear Test)^32^. Using the terminology by Ibbotson and Morton, the term ‘dominant’ will henceforth be applied to combinations going with the hypothesized hierarchy, and ‘non-dominant’ will be applied to combinations going against it. Assessing how musical expertise affects participants’ abilities to coordinate body parts allowed us to obtain nuanced insight in how perception influences action in the form of interlimb coordination and dual motor tasks.

## Methods and materials

### Participants

63 participants were recruited for the study. Three were excluded, as they were unable to perform the rhythmic patterns in isolation. This left 60 participants: Twenty professional musicians (mean age = 23.8, SD = 2.40, 7 female), twenty-two amateur musicians (mean age = 24.36, SD = 1.68, 13 female) and eighteen non-musicians (mean age = 25.06, SD = 3.94, 12 female). The professional musicians were actively performing professionals or conservatory students, the amateur musicians performed music at a hobby level, i.e., on a regular basis for enjoyment, and the non-musicians had no more than one year of formal music education besides the mandatory music classes in primary school. All participants were right-handed as assessed by the Edinburgh Inventory^33^. No attempt was made in order to balance out for gender, since former studies have shown that musical competence generalizes across gender^32^. The study was performed at Center for Music in the Brain, Dept. of Clinical Medicine at Aarhus University in accordance with the guidelines and regulations for behavioral studies in force at Aarhus University. No personal sensitive information was solicited from participants, and informed consent was obtained for all participants at the beginning of each session.

### Paradigm

**Figure 2:**
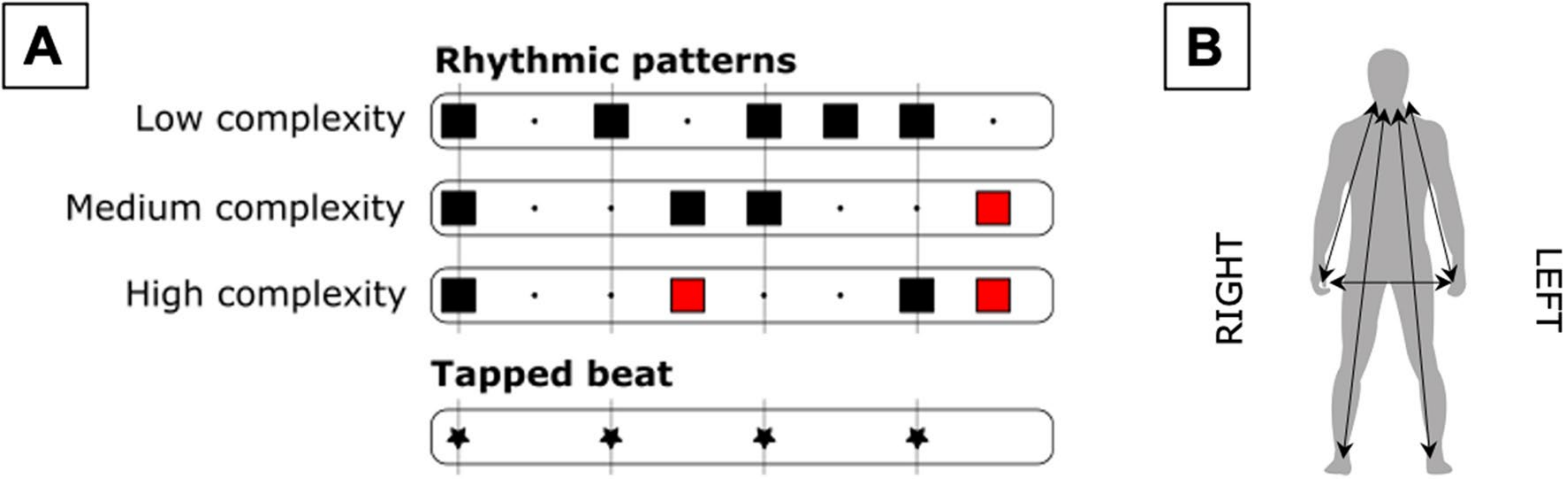
Experimental stimuli and conditions. (**A**) Transcription of the rhythmic patterns used for the three subtests. (**B**) The effector combinations used in the experiment: V + RH, V + LH, V + RF, V + LF, RH + LH, and RH + LF, each in their dominant and non-dominant version respectively. Syncopes are highlighted in red to emphasize the difference in difficulty. Figure was created using Inkscape 1.1 (https://inkscape.org), Adobe Illustrator 2018 22.0.0 (https://www.adobe.com/products/illustrator.html) and Microsoft Word for Mac, version 16.49 (21,050,901) (https://www.microsoft.com/da-dk/microsoft-365/word).

The experimental paradigm consisted of three subtests, each with a different rhythmic pattern assigned to it (Fig. 2). The rhythmic patterns were designed to be of different complexity (low, medium and high), based on the amount of syncopes (when a strong beat is not articulated, but is preceded by an articulated weak beat) 4/30/2022 7:31:00 AM. The participants’ task was to produce the rhythmic pattern of the given subtest using one effector while maintaining a regular beat with another effector. While the hands and feet produced sound by tapping, the participants were instructed to vocalize the syllable ‘da’, when using their voice. Participants used combinations of five different effectors to perform the rhythm/beat tasks: voice (V), right hand (RH), left hand (LH), right foot (RF) and left foot (LF). Henceforth, when referring to a combination pair, a plus sign will be used, i.e., RH + LF.

The combinations including the voice were of primary interest, as all combinations of hands and feet had already been studied previously^25^. For the purpose of replication, we also tested RH + LH and RH + LF. Thus, 6 combination pairs were included: V + RH, V + LH, V + RF, V + LF, RH + LH, and RH + LF, each in their dominant and non-dominant version respectively.

### Procedure

First, participants were informed that the purpose of the experiment was to assess their ability to perform a rhythm with one part of their body while keeping the beat with another. A training session preceded each subtest, ensuring that participants were able to perform the rhythm with each effector in isolation, so that differences in performance would not be due to motor deficiencies. First, the experimenter demonstrated the rhythmic pattern to the participant, until they were familiar with it. Then, the experimenter tapped the beat, while the participant performed the rhythmic pattern consecutively with each effector. The training session ended when the participant felt comfortable with the rhythmic pattern and performed it correctly with all effectors, as assessed by the experimenter.

In the actual experiment, the order of the subtests was the same for all participants (low–high–medium), but two different orders of the combinations were counterbalanced between the participants (limb combinations are written as rhythm/beat): Order 1: V/RH, V/LH, V/RF, V/LF, RH/V, LH/V, RF/V, LF/V, RH/LH, LH/RH, RH/LF, LF/RH. Order 2: V/LF, V/RF, V/LH, V/RH, LF/V, RF/V, LH/V, RH/V, LH/RH, RH/LH, LF/RH, RH/LF. Order 3: RH/LH, LH/RH, RH/LF, LF/RH, V/RH, V/LH, V/RF, V/LF, RH/V, LH/V, RF/V, LF/V. Order 4: LH/RH, RH/LH, LF/RH, RH/LF, V/LF, V/RF, V/LH, V/RH, LF/V, RF/V, LH/V, RH/V.

To ensure that the tempo of the performances would be comparable, the participants wore headphones with a click track playing a regular beat at 90 bpm (i.e., with a beat periodicity of 667 ms). The volume was adjusted to a comfortable level before the experiment, and they were instructed to make their combined performance correspond to the tempo of the click track.

The participants had two trials per combination and they did not receive any feedback during the experiment. Once the click track started, they were allowed a few measures to adjust to the beat before and between trials. They were also allowed to start tapping the beat a few measures before performing the rhythmic pattern. It was emphasized, though, that they were not allowed to practice with both effectors before or between trials.

To measure rhythmic competence, the participants subsequently completed the rhythmic part of the Musical Ear Test^19^. The test consists of 52 pairs of short rhythmic phrases, and the participants judged whether the pairs were identical or not. The test is auditory only and thus gives a measure of the participants' perceptive abilities.

### Equipment

All trials were audio recorded in stereo with one microphone for the rhythm effector and one for the beat effector. The microphones were moved according to the active effectors during the trial in question. One microphone was handheld by the experimenter, while the other was mounted on a stand. A TC Electronics Konnekt 8 soundcard and the program Reaper 64 were used for the recordings.

### Evaluation

The participants performed each combination twice. The recording of each combination was pseudo-anonymized and then evaluated by both an experimenter and an external rater, educated at a music conservatory. Both performances were evaluated and the best was selected for analysis. The two raters were blind to the effectors used, besides the voice.

Each combination was given a grade between 1 and 5 from the following principles: (1) the combination was not accomplished, (2) the combination was almost accomplished, but with some mistakes, (3) the combination was accomplished, but somewhat imprecise in tempo, (4) the combination was accomplished, but one or two beats were imprecise in tempo, and (5) the combination was accomplished without mistakes. The final score for each combination was the average of the grade given by the experimenter and the external reviewer. This will be referred to as the Performance Score.

### Inter-rater reliability

Inter-rater reliability between the two raters was calculated using an average-measures, absolute-agreement, two-way mixed-effects model^34^. The resulting intra-class correlation (ICC) was within the excellent range, ICC = 0.97 (95% CI [0.953;0.979], p < 0.001), indicating that the raters had a high degree of agreement. The high ICC suggests that a minimal amount of measurement error was introduced by the two independent raters, and therefore statistical power for subsequent analyses is not substantially reduced. An average of the two raters’ evaluation was therefore deemed to be a suitable score for use in the hypothesis tests of the present study.

### Statistical approaches

The average Performance Score was confounded by a large number of participants, mainly musicians, who were able to complete almost all combinations equally well. Specifically, 22 participants (13 professional musicians and 9 amateur musicians) obtained an average Performance Score of 4.9 or above in either the dominant or the non-dominant combinations, or both. Hence, two approaches to the analysis were carried out: One using the Performance Score as the outcome variable, but excluding the 22 ceiling performers, leaving 38 for analysis; and one using the binomial outcome variable Accuracy, that was derived from the Performance Score, indicating whether a combination was accomplished or not. Performance Scores of 2.5 and above were deemed as accomplished. All participants were included in these analyses.

Initially, comparisons of Accuracy in the non-dominant and dominant version were made at each different effector combination using the mid-p version of McNemar’s exact conditional test for paired binary observations^35^, to make an initial assessment of the hierarchy. Then two different models, each with a different format, were constructed: The first one tested the hypothesis that the voice occupies the top of the hierarchy by fitting a generalized linear mixed-effects model (GLMM) on the binary outcome Accuracy. The combinations included in this model were limited to those involving the voice. The fixed factors included in this analysis were (1) **Group** (between-subject: Non-musician, amateur musician and professional musician): Increased Accuracy for amateur musicians and professional musicians respectively is expected as compared to non-musicians, (2) **Rhythm** Complexity level (within-subject: low, medium and high): Increased Rhythm Complexity is expected to affect Accuracy negatively, and (3) **Direction** (within-subject: dominant or non-dominant): Increased Accuracy for the dominant combinations is expected and would confirm the hypothesized hierarchy. Random intercepts for participants were included as well as by-participant random slopes for the effect of direction, which accounted for inter-individual differences in the effect of working against the hierarchy. The second tested the hypothesis that the effect of direction depended on the expertise level of the participants by constructing a linear mixed-effects model (LMM) including also non-voice combinations, and fitting it on the average Performance Score of the remaining 38 participants. Due to the vast reduction of the groups of professional musicians and amateur musicians, the group factor was replaced by the MET-score, which served as an index of musical expertise. Accordingly, the fixed factors in this analysis were (1) **MET-score** (between-subject: continuous score from 1 to 52): An increased MET-score is expected to affect Performance Score positively, (2) **Rhythm** Complexity level (within-subject: low, medium and high, medium and complex): An increased level of rhythm Complexity is expected to affect the average Performance Score negatively, and (3) **Direction** (within-subject: dominant or non-dominant): An increased Performance Score for the dominant combinations is expected and would again confirm the hypothesized hierarchy. The random effects were similar to the GLMM’s constructed previously. The possibility of an interaction effect between MET-score and direction was of particular interest in this model.

The analyses were conducted using the lme4 package^36^ in the statistical software R (version 3.4.4, R Core Team, 2018) with RStudio (version 1.1.456, RStudio Team, 2016). ﻿The statistical significance level was set at p < 0.05.

To assess tapping data more closely, we extracted the exact tapping onsets of three representative subjects; one professional musician, one amateur musician and one non-musician. Onset estimation was done automatically with additional auditory and visual tracking, which was necessary due to the noise in the audio data. For each accomplished trial, we calculated the mean absolute asynchrony between expected and observed taps for both rhythm and beat. For trials not accomplished, we assessed the different error types and listed them for each participant. For these trials, mean absolute asynchrony could not be calculated.

### Control experiments

Two control experiments were performed after the original data collection. Control experiment 1 used 18 right-handed professional musicians from the original sample to confirm the hierarchical organization of hands and feet by self-assessment and to control for the possibility of the hierarchy only pertaining to the three rhythms used in this study. The musicians’ task was to keep the beat with one effector and improvise rhythmically with another, in dominant and non-dominant versions of the combinations V + RH, RH + LH, LH + RF and RF + LF. The order of the combinations was counterbalanced between subjects. After each combination pair, participants were asked if they found the dominant or non-dominant version easiest.

**Figure 3:**
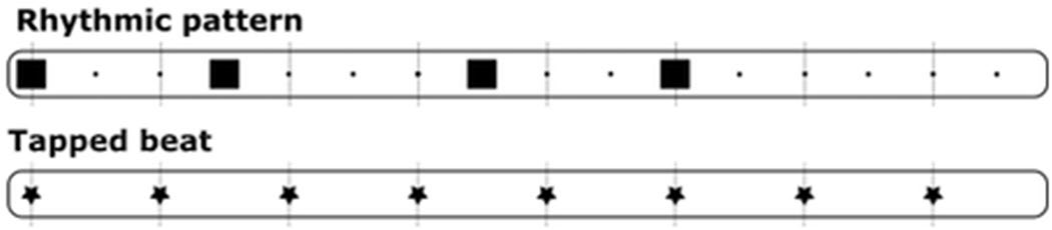
Rhythmic pattern used in control experiment 2. Figure was created using Inkscape 1.1 (https://inkscape.org).

Control experiment 2 used 19 right-handed professional musicians from a different sample in connection with a separate study to control for the possibility of the hierarchy being caused by dexterity differences between effectors, i.e., single-effector dexterity. The participants performed a rhythmic pattern and its corresponding beat in both directions of the combination pairs Clap + RF, V + Clap and RH + LH. This rhythmic pattern had longer IOI’s than the corresponding beat (Fig. 3). After each trial, the musicians were asked to rate how easy they found the task on a scale from 0 to 100. The order of the combinations was counterbalanced between subjects.

## Results

The difference between Accuracy in the dominant and non-dominant versions of each effector combination were highly significant in all but the RH + LH condition; here, only the high complexity rhythmic pattern yielded a significant difference (Table 1), while the others only showed a tendency.

**Table 1:** Mid-p version of McNemar’s exact conditional test of the difference between dominant and non-dominant version of the different effector combinations. Table shows p-values of each test.

Combination	Low complexity	Medium complexity	High complexity
V + RH	< 0.001	< 0.001	< 0.001
V + LH	< 0.001	< 0.001	< 0.001
V + RF	< 0.001	< 0.001	< 0.001
V + LF	< 0.001	< 0.001	< 0.001
RH + LH	0.07	0.55	0.008
RH + LF	0.004	< 0.001	< 0.001

The general linear mixed-effects model based on the voice combinations confirmed that voice is placed at the top of the hierarchy, in that the Direction affected Accuracy significantly χ^2^ (1) = 76.145, p < 0.001. Performing the dominant combinations gave participants a 7.47 higher log odds of completing compared to the non-dominant combinations (z = 8.12, p < 0.001).

**Figure 4:**
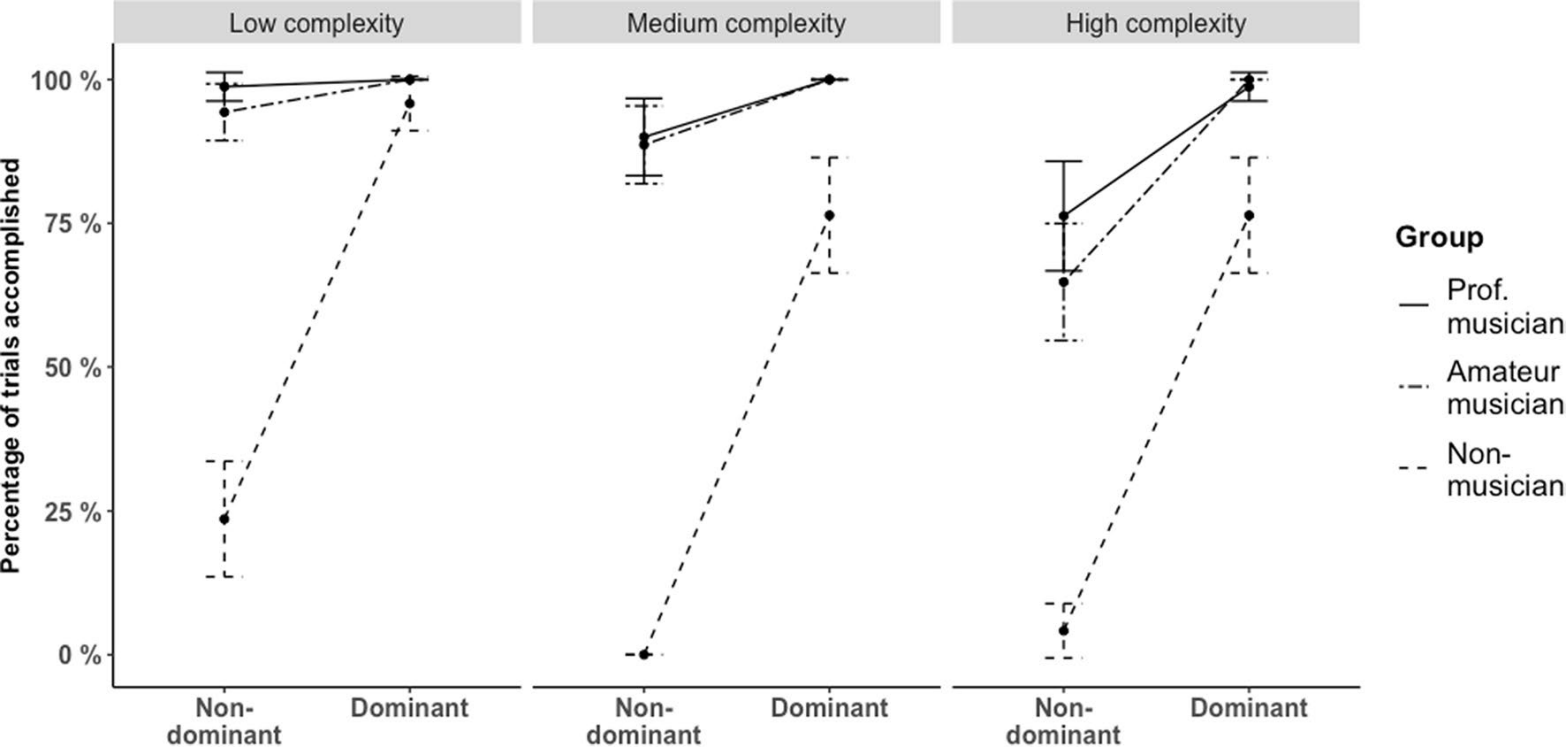
The percentage of accomplished trials. Split in subtests and expertise groups, showing only combinations including the voice. Vertical bars represent 95% confidence intervals.

Accuracy was also affected significantly by both Complexity, χ^2^ (2) = 122.86, p < 0.001, and Group, χ^2^ (2) = 62–01 p < 0.001, as expected. Planned contrasts in the Group variable showed that compared to the amateur musicians, the log odds of completing were significantly lower for non-musicians (*b* = − 6.59, *z* = − 7.12, p < 0.001), and higher, but not significantly, for professional musicians (*b* = 0.63, *z* = 0.74, p = 0.457). Planned contrasts in the Complexity variable showed that compared to the medium complexity rhythm, the log odds of completing were significantly lower for the high complexity rhythm (*b* = − 1.27, *z* = − 4.05, p < 0.001), and significantly higher for the low complexity rhythm (*b* = 2.68, *z* = 6.12, p < 0.001). See Fig. 4. There was no significant interaction effect of the MET-score and Direction.

**Figure 5:**
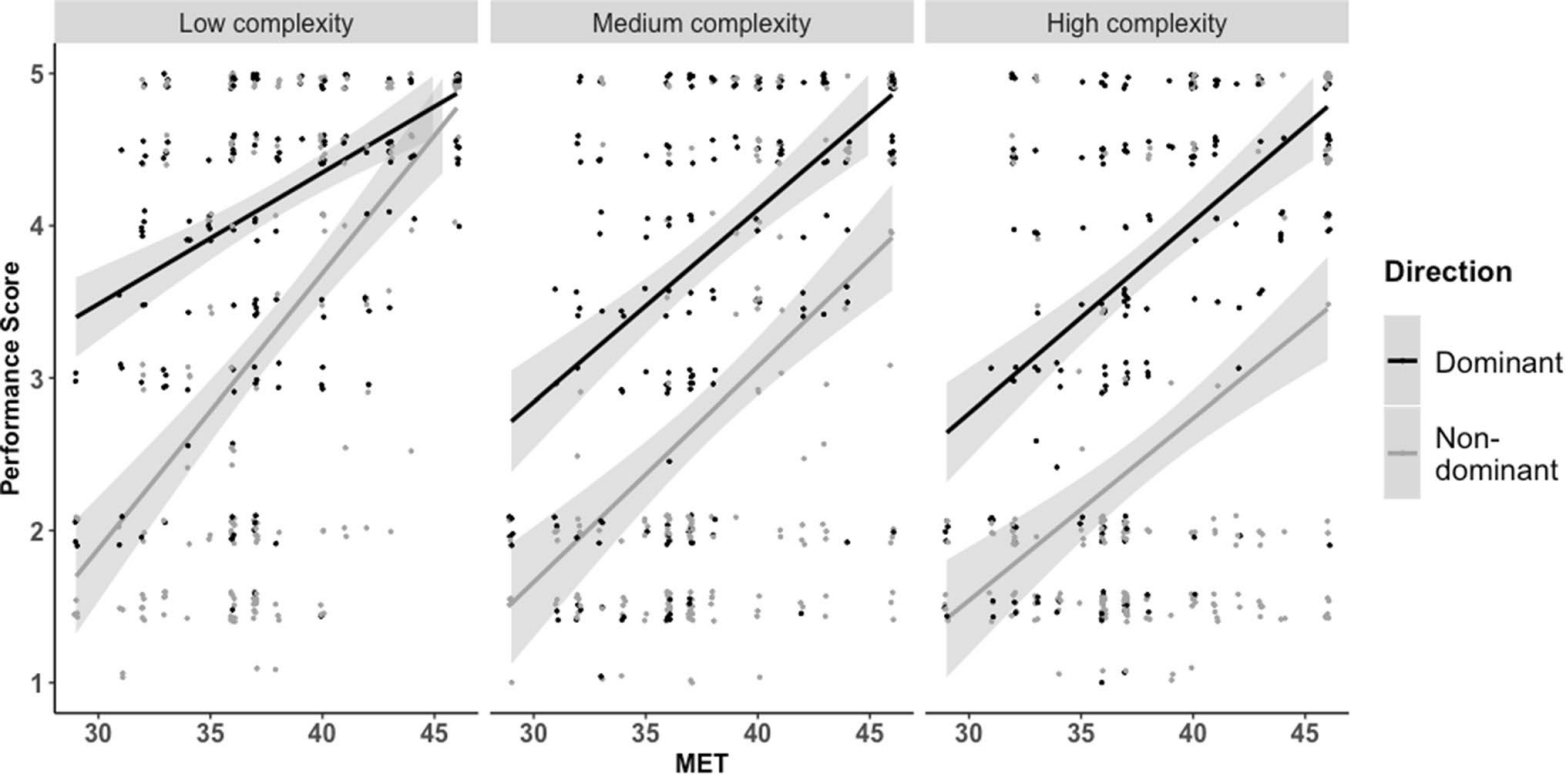
Performance Score as a function of MET-score. The chart is divided in subtest and direction of combinations. 95% confidence intervals are shown.

MET-score affected the average Performance Score significantly, χ^2^ (2) = 20.421 p < 0.001, in that 1 MET-point increased the average Performance Score with 0.17 points ± 0.03 (standard error). Direction still affected the Performance Score, χ^2^(2) = 16.14, p < 0.001 with the dominant combinations having a 2.79 points ± 0.89 (standard error) higher average Performance Score than the non-dominant combinations. Complexity also affected the Performance Score significantly, χ^2^(2) = 139.47, p < 0.001, with the low complexity rhythm leading to 0.44 points higher ± 0.06 (standard errors), and the high complexity rhythm leading to 0.19 points lower ± 0.06 (standard errors), respectively, compared to the medium complexity rhythm See Fig. 5.

**Figure 6:**
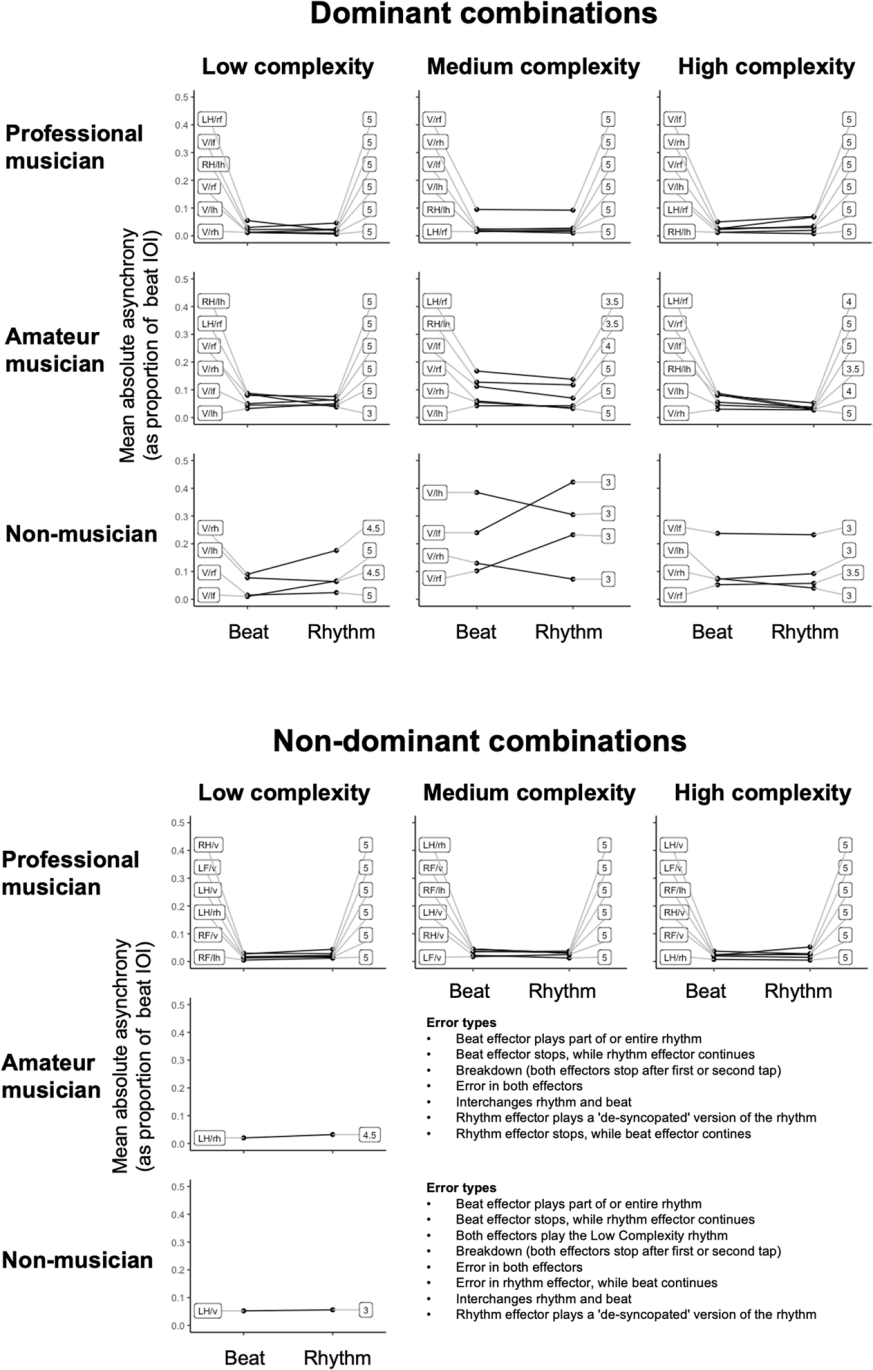
Mean absolute asynchrony as proportion of beat inter-onset interval for completed trials, based on one subject from each expertise group. Labels within plots show combination type (left) and Performance Score (right). For the amateur and non-musician, no plots are shown for the non-dominant medium and high complexity trials, as they failed to accomplish any trials in these conditions. Descriptions of error types in unsuccessful trials are displayed here instead.

Figure 6 shows an overview of the tapping data of the three representative participants, along with a description of the error types for the trials which were not accomplished. The plots show that the non-musician generally has a higher mean absolute asynchrony than the amateur musician and professional musician. The non-musician and amateur musician accomplished fewer trials in the non-dominant combinations compared to the musician—in fact, they both failed to complete any trials in the medium and high complexity conditions. Error types do not show a clear pattern of a breakdown of either the rhythm or the beat, but differ across trials.

### Control experiment 1

**Figure 7:**
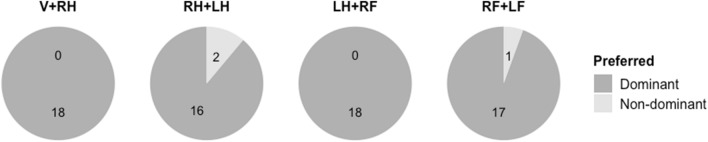
Distribution of preferred combinations among professional musicians. Out of 18 musicians, the dominant combination was preferred by 18 in V + RH, 16 in RH + LH, 18 in LH + RF and 17 in RF + LF.

Results convincingly confirm the proposed hierarchy and are seen in Fig. 7. Out of 18 professional musicians, the dominant combination was preferred by 18 in V + RH, 16 in RH + LH, 18 in LH + RF and 17 in RF + LF.

### Control experiment 2

For each combination pair, a paired t-test was conducted to compare the dominant and non-dominant combinations. There was a significant difference between the scores of Clap/RF (M = 82.53, SD = 19.30) and RF/Clap (M = 62.05, SD = 23.93); t(18) = 4.08, p < 0.005, as well as between the scores of V/Clap (M = 86.84, SD = 10.70) and Clap/V (M = 59.53, SD = 21.16); t(18) = 5.8987, p < 0.005). Thus, the results of the combinations of non-homologous effectors (Clap + RF and V + Clap) were similar to the results already presented in this study. The same difference was not found between the combinations RH + LH. However, a paired t-test on the difference in ratings between the group that tried the dominant version of the combination pair first (M = 13.33, SD = 14.58) and the group that tried the non-dominant version first (M = − 6.56, SD = 9.55) showed a significant difference between the two groups, t(13.802) = 3.42, p = 0.004, indicated that the subjects generally preferred the combination, they tried first.

## Discussion

We present data supporting the existence of the proposed comprehensive bodily hierarchy governing interlimb coordination between voice, hands and feet during simultaneous performance of rhythm and beat. From lower to higher levels, the hierarchy incorporates (1) left foot, (2) right foot, (3) left hand, (4) right hand, (5) voice. Participants’ execution of the musical rhythm/beat tasks was more precise when the supporting role of keeping the beat was undertaken by an effector occupying a lower level in the hierarchy than the effector performing the rhythm. In combinations of hands and feet, we observed better performance when beat-keeping was assigned to a foot while the rhythm was performed using the hands. Yet, when the supporting role of beat-keeper was assigned to the voice, it was strikingly difficult for the participants to perform the combination with the rhythmic pattern in the hands, as indicated by significantly better performance in the opposite combination. In bimanual executions of the task, we observed better performance when beat-keeping involved the left than the right hand in the high complexity rhythmic pattern. This difference, however, was only significant with the high complexity rhythmic pattern.

**Figure 8:**
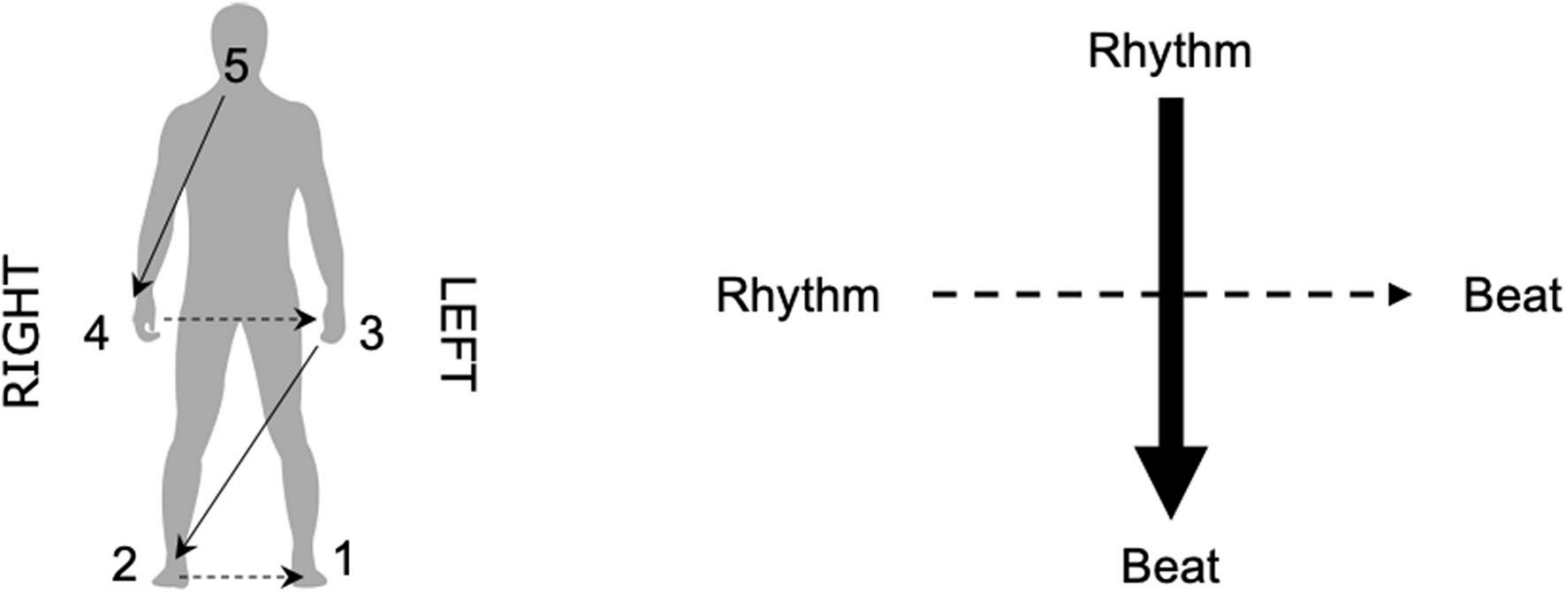
The bodily hierarchy. The bodily hierarchy pertaining to right-handed participants encompasses the following components: (1) left foot—(2) right foot—(3) Left hand—(4) Right hand—(5) voice. As indicated by the arrows, the hierarchy has a horizontal and a vertical dimension of which the vertical dimension precedes the horizontal. Figure was created using Adobe Illustrator 2018 22.0.0 (https://www.adobe.com/products/illustrator.html) and Microsoft Word for Mac, version 16.49 (21,050,901) (https://www.microsoft.com/da-dk/microsoft-365/word).

While performance generally increased with musical expertise, this hierarchical pattern was consistent across groups of non-musicians, amateur musicians, and professional musicians. A subsequent control experiment in which the rhythm pattern had longer IOIs than the corresponding beat pattern ruled out the possibility that dexterity accounts for the results and suggested a precedence of the vertical over the horizontal axis. A follow-up experiment in which participants improvised with one effector while keeping a steady beat with another confirmed the bodily hierarchy and extended the results to improvisational behavior. Taken together, our results suggest a comprehensive bodily hierarchy of interlimb coordination with a vertical axis preceding a horizontal axis as illustrated in Fig. 8.

By using musical rhythm, we were able to create coordination tasks including two separate actions (performing rhythm and keeping the beat) that could be performed on equal terms by hands, feet and voice, thereby allowing the roles of the effectors to interchange. Previous studies on verbal/manual coordination have used either fundamentally different tasks for voice and hands, for instance speaking and tapping^29,37,38^, or exactly similar tasks, i.e., identical rhythmic patterns with the body and voice^30,39^. These studies found interference due to dual-tasks in the first examples and mutual stabilization of the two body parts in the latter.

According to Kinsbourne and Hicks^27^, performing two different tasks simultaneously without losing efficiency on the main task requires a high degree of automatization in one of the tasks. Our study, which is based on the musicological dichotomy between rhythm and meter/beat, revealed that this assumption depends on which effector performs which action. The possible stabilizing effect^39^ only appears if the supporting role, i.e., the beat, is maintained by a lower level of the bodily hierarchy while a higher level maintains the conducting role, i.e., the rhythm. Even to musicians, to whom beat-keeping is highly automatized, the task of performing a rhythmic pattern with one hand was complicated by having the voice executing the task of keeping a beat. This happened despite the fact that the vast majority of the musicians were perfectly able to perform the opposite dominant combinations.

Musicians generally excel in performing both simple rhythms, complex rhythms^20^, and polyrhythms^40–42^. In the present study, musicians similarly outperformed non-musicians, and Performance Scores generally increased with performance on rhythm perception tasks (the Musical Ear Test). Importantly, the difficulties for the low-scoring participants of our study did not arise during simple tapping or vocalization of the rhythm in isolation, as all participants were able to perform the rhythm and beat separately in the training session. Rather, the challenges arose when *combining* the two components. As evident by Fig. 6, the error types were heterogeneous, differing between errors in rhythm, errors in beat, complete breakdown of both effectors, etc. Exploring putative patterns in error types and in particular their relation to musical expertise more comprehensively could be a relevant topic for further study. Previous studies^43^ have shown that when learning to tap 3:2 polyrhythms, integrated training is more effective than training the hands separately, indicating that learning to tap a bimanual polyrhythm requires that the participants view the left- and right-hand rhythms as one single action as opposed to combining two separate actions. Consistently with this, we may speculate that the musicians in our study excel at coordinating rhythm and beat, because they perceive it as one unified action.

Previous studies have indicated that a hallmark of musical expertise is musicians’ ability to form more precise musical predictions^6,44^. These studies were inspired by influential theories of brain function—also known as predictive processing^45^—positing prediction as the fundamental principle behind brain function. These theories are based on physiological observations^46,47^ and offer integrative accounts of perception and action^48–51^. Conceptually, rhythm can be described as the acoustical input to our ears, whereas the meter is the brain’s posterior expectations that constitute its predictive model. The rhythm can be more or less conflicting with the meter, creating stronger or weaker prediction error between auditory input and predictive model^52^.

Studies on musical groove have shown that high rhythmic expertise corresponds to a strong predictive model (the meter), which results in less destabilization when synchronizing one’s body to the beat of a syncopated groove^53^. Hence, the more precise predictive model makes it easier for musicians to attenuate prediction errors than non-musicians^54^. This ability is however still less pronounced in the non-dominant combinations. Control experiment 2 was inconsistent with single-effector dexterity as an explanation of the vertical part of the bodily hierarchy, since the musicians in this experiment—even with a rhythmic pattern with longer IOI’s than the beat—still preferred to keep the beat with an effector lower in the hierarchy than the effector performing the rhythm. Instead, the bodily hierarchy seems to be an abstract organization of conducting and supporting roles—conceptualized in music as the tension-creating dichotomy between rhythm and meter—which changes precision-weighted prediction error that shifts dynamically according to where in the body it takes place.

The hierarchical organization of brain responses described by theories of predictive processing maps well onto the hierarchical structures of musical rhythms^14^. While expectations and predictions are assumed specific to different species and formed by individuals’ environments, the theories of predictive processing do not in themselves offer accounts of these environmental constraints which impact on perception and behavior^55^ (for a critical review of its limitations, see Heilbron & Chait^56^). Ecological theories, on the other hand, emphasize the reciprocity between living organisms and their physical environments which plays a role in the organisms’ phylogenetic and/or ontogenetic development.

Taking environmental constraints into account, Bernstein^26^ approached the problem of the abundance of degrees of freedom in motor coordination and how the central nervous system chooses the solution which leads to the most dexterous performance. This framework of flexible hierarchical control was recently proposed as a unifying framework for divergent ideas of synergies^57^ (and it is relevant in the present context where we essentially asked participants to suppress the “best solution” in the non-dominant conditions. The asymmetry which is specific to level D, the level of action, could be key to explaining the relative difficulty for participants of going against the horizontal plane of the proposed bodily hierarchy. The vertical part, in turn, may be rooted in the fundamentally different kinds of dexterity that are necessary for keeping a stable beat (*body dexterity,* subserved by level B and C), and performing a rhythm (*hand and object dexterity,* subserved by level D). When combining the two in the dominant combinations, level D takes the leading role, while relying on the automaticity at lower levels B and C, an automaticity which is far more developed in musical experts. The automatism at lower levels is disrupted in the non-dominant combinations, however, where the switched effector roles pose difficulties for musicians and becomes impossible for many non-musicians, consistent with the notion that skill development leads to stronger guards against the disruptive switching of leading levels and subsequent deautomatization of background levels.

Our data show indisputable evidence of the voice occupying the highest level of the bodily hierarchy. With voice as the primary conveyer of language, it is natural to link this hierarchical position to the connection between language and rhythm. This connection has been examined and demonstrated in research on rhythm both as a beat-like, isochronous rhythms and more varied rhythmic patterns with varying IOI's as well as musical-pedagogical practices. Studies have demonstrated shared cognitive resources between tapping a self-produced counter-meter to a music excerpt and language-syntactic processes residing in the left hemisphere^58^, and strong musical rhythm perception has been associated with grammar learning skills in children^59^, whereas impaired rhythm perception can be found in children who stutter^60^. Earlier studies^25^ have speculated that right-hand dominance in bimanual coordination may be linked to a shared left-hemispheric specialization for speech and right hand. In several common pedagogical practices, rhythmic patterns are taught and practiced through phonetic vocalization before performance is transferred to a musical instrument, such as in the South Indian musical style ‘Konnakol’. Here, a rhythm ‘language’ based on vocal imitations of drum sounds functions both as an artform in itself and as a deeply integrated part of the training required to play the Mridangam drum^61^. Such practices are built on an experience of rhythms being more easily learned when vocalized. This is in accordance with the superior task performance when participants vocalize rhythms rather than the beat observed in the present study. Evidence from the studies mentioned clearly point to a closer connection between voice, language and rhythm than between rhythm and hands or feet. We have added the voice to the bodily hierarchy proposed by Ibbotson and Morton^25^. This addition to the hierarchy is not necessarily exhaustive, though. In other words, we cannot rule out the possibility that a sixth or seventh level of the bodily hierarchy exists above the voice. Future studies are needed to explore this possibility further.

The raters based their evaluation on the raw audio recordings. They were not made explicitly aware of the effectors used, yet it was clear from the recordings which combinations included the voice. We cannot rule out the possibility of a bias in these cases. Additionally, the rhythms of the three subtests were sometimes too easy for the musicians and too difficult for the non-musicians—a well-known challenge in studies on rhythm performance. In some cases, we observed a ceiling effect in the groups of professional musicians and amateur musicians and almost a floor effect in the group of non-musicians. However, the sample size allowed us to exclude the ceiling effect cases and still obtain a valid result with the linear mixed-effects model by omitting the group factor in favor of using the MET-score as an expertise measure. By using MET-scores in addition to the dichotomous categorization of musicianship, we obtained a nuanced view on how musical expertise influences the coordination of rhythm and meter on a group level as well as on an individual level. However, an adaptable design would be advantageous in future studies, where the Complexity level of each rhythm would depend on the performance of the previous rhythm. An adaptable paradigm may also reveal a difference between professional musicians and amateur musicians, which did not appear in the present study, where the best musicians’ true potential could not be realized.

Overall, this study suggests a comprehensive bodily hierarchy with the voice occupying the highest level, showing how interlimb coordination is governed by a hierarchical organization of effectors, reflecting an abstract universal organization of conducting and supporting roles.
